# Epidemiological Assessment of Lumpy Skin Disease in Cattle: Risk Factors at Farm and Animal Levels in Southern Bangladesh

**DOI:** 10.1155/tbed/7813523

**Published:** 2026-05-20

**Authors:** Ibrahim Khalil, Abu Sayed, Mohammed Shahriar Hossain Meraz, Md. Atikur Rahman, Mohammad Eliyas, Md. Jahirul Islam, Mas-ud-hassan, Md. Nurul Alam, Md. Ahasanul Hoque, Najmul Haider, Michael P. Ward

**Affiliations:** ^1^ Field Disease Investigation Laboratory, Department of Livestock Services, Barishal, Bangladesh; ^2^ Faculty of Animal Science and Veterinary Medicine, Patuakhali Science and Technology University, Patuakhali, Bangladesh, pstu.ac.bd; ^3^ Upazila Livestock Office and Veterinary Hospital, Department of Livestock Services, Patuakhali Sadar, Patuakhali, Bangladesh; ^4^ Upazila Livestock Office and Veterinary Hospital, Department of Livestock Services, Chandanaish, Chattogram, Bangladesh; ^5^ Government Duck Rearing Unit, Department of Livestock Services, Jhalokathi, Bangladesh; ^6^ Department of Medicine and Surgery, Faculty of Veterinary Medicine, Chattogram Veterinary and Animal Sciences University, Mymensingh, Bangladesh, cvasu.ac.bd; ^7^ School of Life Sciences, Keele University, Keele, Staffordshire, UK, keele.ac.uk; ^8^ Sydney School of Veterinary Science, University of Sydney, Sydney, New South Wales, Australia, sydney.edu.au

**Keywords:** cattle, communal grazing, lumpy skin disease, risk factors, Southern Bangladesh, vector control techniques

## Abstract

Lumpy skin disease (LSD) is a transboundary viral disease of cattle that has spread from Africa and the Middle East to Asia, including Bangladesh, causing substantial economic loss. We aimed to identify risk factors associated with LSD in cattle and cattle herds in Barishal, Patuakhali, and Chattogram districts of Southern Bangladesh. A total of 6624 cattle from 1260 herds, including those with current (*n* = 194) and past cases (*n* = 445), were included. Diagnosis was based on clinical signs, and farms were selected by convenience sampling. Data on management, environmental, and animal‐level factors were collected via structured questionnaires. Risk factors were analyzed using cross‐sectional (herd‐level) and case–control (animal‐level) designs, with univariate and multivariate logistic regression models applied. Moran’s I statistic was used to assess spatial clustering. The herd‐level prevalence was 15.3% (194/1260), and the animal‐level prevalence was 13.1% (867/6624). High morbidity was observed in young cattle (8.7%; *n* = 315), local breeds (7.7%), and females (7.3%). Multivariate analysis at the herd level showed that the highest case rates were associated with communal grazing (aOR = 1.7), manure disposal in ponds (aOR = 2.1), and reporting the use of chemical sprays (aOR = 1.9), compared to no communal grazing, disposal in pits, and the use of mosquito nets. Local breeds (aOR = 2.7), poor body condition (aOR = 2.2), and communal grazing (aOR = 9.9) exhibited higher odds of infection compared to crossbreeds, good body condition, and no communal grazing. Spatial analysis showed geographic heterogeneity but no significant clustering.(Moran’s index = 0.25; *p* = 0.409). These findings emphasize the need for targeted control measures, including improved manure management and vector control, such as insecticide‐treated nets.

## 1. Introduction

Lumpy skin disease (LSD) is a highly contagious viral infection of cattle caused by lumpy skin disease virus (LSDV), a member of the genus Capripoxvirus [1]. Transmission occurs primarily through blood‐feeding arthropods such as mosquitoes, biting flies, and ticks, although infected cattle also shed virus in skin lesions and bodily secretions, contributing to environmental contamination [1]. Clinically, LSD is characterized by fever, anorexia, weakness, lymphadenopathy, and the development of characteristic firm skin nodules, which may extend to mucous membranes and, in severe cases, involve internal organs [1, 2]. These lesions can progress to edema, necrosis, and ulceration, predisposing affected animals to secondary bacterial infections and, occasionally, death [2]. Additional signs such as lameness, reluctance to move, and swelling of the limbs and brisket may also be observed as the disease advances [2]. Morbidity within herds varies widely (3%–85%), while mortality is generally low (<3%) but may exceed 40% during severe outbreaks [3–5]. Recovery typically takes ~30 days [6]. In low‐resource settings, infected animals are often sold or slaughtered at reduced prices, and movement through markets and abattoirs can unintentionally facilitate disease spread via direct contact and vector exposure [7].

LSD, historically prevalent mainly in African countries, has now become a major concern for European nations as reported cases continue to rise [8, 9]. The disease was first reported in Asia, including Bangladesh, in 2019 and subsequently spread to China, India, Nepal, Taiwan, Bhutan, Vietnam, Hong Kong, the Republic of Korea, and Indonesia by 2020 [4, 10]. In Bangladesh, animal‐level prevalence varied widely across districts during 2019–2020, ranging from 4% in Barishal to over 70% in Rajshahi, with intermediate levels reported in Sylhet, Mymensingh, Gaibandha, and Chattogram, reflecting extensive geographic spread [4, 11–15]. This widespread transmission has caused substantial economic losses due to reduced milk yield, hide damage, impaired fertility, cattle mortality, increased treatment costs, and trade and movement restrictions across the livestock sector [4, 11–15].

LSD is influenced by several risk factors at both the farm and animal levels, which can vary depending on environmental conditions, farm management practices, and animal characteristics [16]. At the farm level, factors such as farm size, herd density, and biosecurity measures play a crucial role in disease transmission. Farms with larger herd sizes and poor biosecurity protocols, including inadequate vaccination programs and limited movement controls, are more likely to experience higher transmission rates [7]. Communal grazing further increases exposure by enabling direct contact between cattle from multiple herds, facilitating cross‐farm transmission [17]. Floor type also contributes to risk as earthen or muddy floors retain moisture and organic matter that promote breeding of biting insects and persistence of pathogens [18]. Similarly, unsafe manure disposal practices, particularly dumping in open fields or ponds, create favorable conditions for vector proliferation and environmental contamination, thereby increasing infection risk [19]. In addition, farms located in regions with high mosquito densities or inadequate vector control are at greater risk due to the vector‐borne nature of LSDV transmission [2].

At the animal level, susceptibility to LSD is influenced by age, immune status, and nutritional deficiencies, with younger cattle, those with compromised immune systems, and animals in poor health or under stress being more vulnerable to infection [5]. Overcrowding and prolonged close contact further enhance transmission by increasing exposure to both infected animals and vectors [4]. Together, these factors highlight the importance of husbandry practices and environmental hygiene in reducing LSD risk. This study aims to identify specific risk factors for LSD at both the farm and animal levels to inform targeted disease control strategies. By examining animal characteristics, management practices, biosecurity, and environmental conditions, the study seeks to improve understanding of LSD transmission dynamics in Southern Bangladesh. Ultimately, the findings are intended to support evidence‐based policy development, improve disease management, and reduce the economic burden of LSD on cattle production systems. We hypothesized that communal grazing and disposal of manure in ponds or open fields are associated with higher odds of LSD infection.

## 2. Methods

### 2.1. Study Location

**Figure 1:**
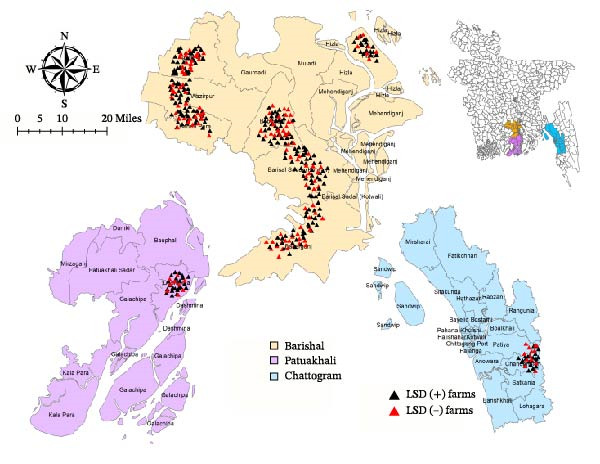
Distribution of lumpy skin disease (LSD) outbreaks at the farm level in Southern Bangladesh (2023). Each dot represents a cattle farm: red = farms with active LSD cases and green = farms without LSD at the time of survey. District and subdistrict boundaries are shown for spatial reference. The map includes a scale bar and north arrow. Data reflect outbreaks recorded during the study period.

This study was conducted from September to November 2023 across three districts of Bangladesh: Barishal, Patuakhali, and Chattogram. Barishal and Patuakhali are situated in the Southern region of the country, whereas Chattogram lies in the Southeastern region (Figure 1). These districts were selected based on ongoing LSD outbreak history and the presence of diverse cattle farming practices. To ensure comprehensive data collection, nine subdistricts were included in the study, with seven from Barishal, one from Patuakhali, and one from Chattogram. The selection of subdistricts was based on accessibility, following a convenience sampling approach.

### 2.2. Study Design and Classification Criteria

This study applied a combination of case–control and cross‐sectional approaches to investigate risk factors associated with LSD outbreaks at both farm and individual animal levels. At the farm level, an unmatched case–control approach was implemented (Box 1).

**Box 1: Clinical case definition used for suspected Lumpy skin disease (LSD).**
 A farm and animal were classified as a suspected LSD case if at least one animal fulfilled both lesion and systemic criteria as outlined below:
 
**Lesion criteria (required):**
  • One or more firm, raised nodules measuring 1–7 cm in diameter; and/or
  • Evidence of necrosis, ulceration, or scab formation.
 
**Systemic signs (at least one required):**
  • Fever
  • Enlargement of superficial lymph nodes
  • Limb or brisket edema
  • Lameness or reluctance to move
  • Anorexia or general weakness
 
**Exclusion criteria:**
 Animals presenting with dermatological conditions inconsistent with LSD (e.g., dermatophilosis, allergic skin reactions, or transient insect bites without firm nodules) were excluded from case classification.
 
**Note:** *Diagnosis was performed by trained veterinarians using standardized criteria across all study districts*.

Due to logistical constraints, laboratory confirmation was not possible for all cases. However, a representative sample of 100 cases was sent to the Central Disease Diagnostic Laboratory under the Department of Livestock Services for routine surveillance, where 22 samples were identified as seropositive for LSD. Therefore, farms were classified based on clinical findings reported during structured interviews. Control farms were defined as those with no recorded LSD cases, either currently or within the past 6 months. None of the animals were vaccinated against LSD. A formal *a priori* sample size calculation was not performed because the study was based on a field outbreak investigation with time‐bound access to farms.

**Figure 2:**
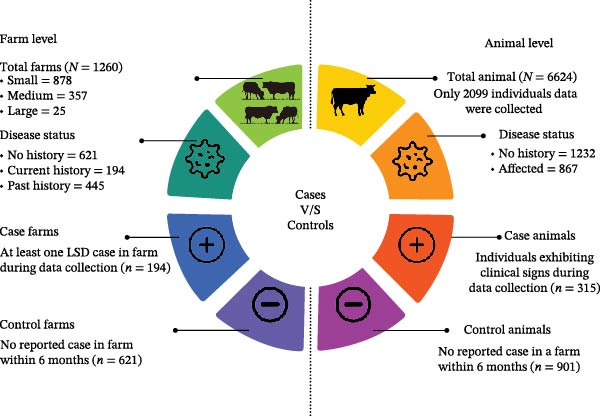
Flowchart of enrollment of clinical lumpy skin disease (LSD) cases and controls, farms and animals, in three districts (Barisal, Patuakhali and Chattogram) of Bangladesh. This flowchart details the selection process for LSD cases and controls at both the farm and animal levels. Case farms are those with ongoing LSD outbreaks at the time of data collection, while control farms had no reported cases in the last 6 months. Case animals exhibited clinical signs of LSD, whereas control animals were healthy and selected randomly from farms with no ongoing outbreaks. The figure shows the enrollment process, illustrating the distribution of case and control farms and animals across different herd sizes, ensuring a clear categorization of participants for subsequent risk‐factor analysis.

A total of 1260 farms were surveyed and categorized based on herd size into small (1–5 animals; *n* = 878), medium (6–15 animals; *n* = 357), and large (16–102 animals; *n* = 25), with a cumulative population of 6624 cattle. Herd‐size categories were defined using data‐driven cut‐off values based on the observed distribution of herd sizes in the study population to ensure meaningful group comparisons and adequate sample size within each category for statistical analysis. Among these farms, 621 had no history of LSD, 194 had ongoing cases during the study period, and 445 had experienced cases within the past 6 months; however, only farms with current cases were included in the risk‐factor analysis at the farm level because farms with past outbreaks might have modified their management and biosecurity practices after the outbreak, so their current exposure status might not reflect conditions at the time LSD occurred. At the individual animal level, a cross‐sectional approach was adopted to evaluate the distribution and potential determinants of LSD. A total of 2099 cattle from the surveyed farms were assessed, comprising 1232 clinically healthy animals and 867 cattle that exhibited characteristic clinical signs of LSD, drawn from farms with ongoing outbreaks and from farms with no history of LSD. On farms with active outbreaks, all clinically affected animals present at the time of visit were enrolled. For the selection of clinically unaffected animals, a complete list of healthy cattle was compiled from each farm’s herd roster, and animals were selected using simple random sampling (lottery method). Where herd size permitted, approximately three unaffected animals were selected for each affected animal. For the animal‐level risk‐factor analysis, we further excluded animals with incomplete exposure data and all animals from farms with only historical cases; after these exclusions, data from 315 affected animals and 901 unaffected animals were analyzed to determine risk factors for LSD infection. An imbalance in the number of case and control farms and animals was observed, with fewer LSD‐positive units compared with LSD‐negative ones. This reflects the natural epidemiological pattern of LSD outbreaks in the study area, where only a proportion of farms were affected at any given time. Additionally, logistical constraints and accessibility influenced recruitment, resulting in a higher number of unaffected farms and animals being enrolled. No artificial matching or oversampling was performed, and all eligible cases identified during field visits were included. Historical cases were utilized solely for prevalence estimation, incidence calculations, and spatial autocorrelation analysis to assess disease distribution trends in the study areas (Figure 2).

### 2.3. Questionnaire Development and Data Collection

To investigate the risk factors associated with LSD, a structured questionnaire was designed to collect data at both the farm and individual animal levels. The initial draft was developed in English and subsequently translated into Bengali to facilitate effective communication with local cattle farmers. Content validity was assessed through expert review by two field veterinarians and one epidemiologist, who evaluated the questionnaire for relevance, clarity, and completeness. A pretesting phase was conducted among 20 randomly selected cattle households in Barishal, and feedback was used to refine question wording, sequencing, and response options. Before data collection, nine veterinarians (government veterinarians and veterinary officers) received a 1‐day training workshop covering study objectives, case definition criteria, interview techniques, ethical considerations, and standard operating procedures for data recording. Mock interviews and field simulations were conducted to ensure consistency across interviewers and minimize observer bias. The veterinarians were selected by the district livestock offices based on their involvement in routine field veterinary services and availability for the study. A convenience sampling method was used, focusing on areas with frequent LSD outbreaks. This nonrandom approach may introduce selection bias and limit the representativeness of the sample as not all outbreak areas were equally represented. Data collection was conducted through direct, face‐to‐face interviews with farm owners or managers responsible for day‐to‐day cattle management. Written informed consent was obtained from all participants before participation, and they were comprehensively briefed on the study’s objectives, confidentiality measures, and their right to withdraw at any stage without consequence. Each interview was designed to last ~15 min, and data were recorded using paper‐based forms. The questionnaire comprised a mix of closed‐ended, semiclosed, and open‐ended questions to capture both quantitative and qualitative insights. For each clinically affected animal, clinical outcomes (recovered, remained ill, sold, or died) were recorded through structured interviews with farm owners or managers during follow‐up at the time of data collection. The outbreak of LSD in Southern Bangladesh has been ongoing throughout the year. Due to the absence of a proper data recording system, it was not possible to track outbreak onset and duration. Additionally, since the outbreak continued beyond the study period, retrospective assessment of its timeline was not feasible.

### 2.4. Variables of Interest

To evaluate potential risk factors, a wide range of epidemiological, environmental, and management‐related variables were considered. Candidate variables were identified based on previously published studies on LSD epidemiology, expert consultation with field veterinarians and epidemiologists, and practical considerations of cattle rearing and environmental conditions commonly observed in the study districts.

At the farm level, data on herd management, environmental conditions, biosecurity measures, and animal movement patterns were recorded. Farms were classified into three cattle‐rearing systems: intensive (cattle remain confined within farm premises), semiintensive (cattle leave the farm for communal grazing but return daily), and extensive (cattle remain outdoors continuously and frequently access communal grazing areas). Farm‐level communal grazing was considered a key variable, reflecting whether a farm allowed its cattle to leave the premises and graze with other herds. Such practices are known to increase exposure to LSD through contact with infected herds and insect vectors at shared grazing and watering sites [20]. Environmental variables included the presence of stagnant water bodies, proximity to dense vegetation, and flooring types (e.g., concrete, mud, or brick with straw), as these factors can influence vector abundance and facilitate pathogen persistence in the environment [21]. Additionally, manure disposal methods and farm hygiene practices, such as cleaning frequency and the use of disinfectants, were documented to evaluate their role in disease prevention. Insect control measures, such as mosquito nets, sprays, and coils, were assessed. During piloting, this question was revised to allow multiple responses and to include the explicit option “no specific vector‐control measure used” to minimize reporting bias and ensure that farms not practicing any insect control could be accurately identified. Information on cattle movement patterns and grazing land use (e.g., marshy lowlands, paddy fields, and drylands) was collected due to their relevance for vector exposure and herd interaction during grazing.

At the individual animal level, factors such as age, breed, body condition score (BCS), and reproductive status were analyzed. Cattle were categorized into three age groups: calves (0–6 months), young cattle (7–24 months), and mature cattle (>24 months) as susceptibility to LSD may vary with age due to immunological differences. Breed classification distinguished between indigenous local zebu‐type cattle and common crossbred types in Bangladesh, primarily crosses between local cattle and Holstein Friesian or Sahiwal [22]. Nutritional status was assessed with BCS, using a standard 1–5 BCS scale [23], with animals classified into poor (BCS 1–2), moderate (BCS 3), and good (BCS 4–5) condition categories. Reproductive status in female cattle was categorized as heifers (precalving females that had never calved), milking cows (lactating at the time of visit), dry cows (nonlactating cows that had calved previously), and pregnant cows to determine potential physiological influences on disease susceptibility. At the individual animal level, exposure to communal grazing was assessed to capture direct risk for each animal, defined as whether the animal had shared a grazing area with other herds during the 2 weeks preceding clinical onset. Even within farms that practice communal grazing, not all animals may participate equally. Animals that actually shared grazing areas with other herds prior to the outbreak might be at higher risk of contracting LSD due to increased opportunities for disease transmission via insect vectors or direct contact.

To ensure statistical rigor, variables with low response rates (<10%), temporal ambiguity, weak biological plausibility, or high collinearity (i.e., variance inflation factor [VIF] > 5) were excluded from the final multivariate analysis. Here, “temporal ambiguity” refers to variables for which the timing of exposure relative to LSD occurrence could not be clearly established (e.g., management practices implemented after an outbreak), whereas “weak biological plausibility” refers to variables without a clearly supported mechanism linking them to LSD susceptibility or transmission.

### 2.5. Statistical Analysis

Descriptive statistics were used to summarize the distribution of LSD cases by demographic variables, including age, sex, and breed. The overall prevalence of LSD was estimated for the total sample (*N* = 6624) and stratified across these demographic categories. Comparisons between demographic groups (e.g., young vs. adult, local vs. crossbred, and female vs. male) were conducted using two‐proportion *z*‐tests, with LSD status (case vs. noncase) as the binary outcome variable. Cluster‐robust standard errors were applied to account for potential intrafarm correlation in animal‐level analysis. Results were reported as percentages with corresponding *p*‐values and 95% confidence intervals (CIs). *χ*‐square tests were used to assess the association between farm LSD status (outbreak vs. no outbreak) and subdistrict, and the results were presented with odds ratios (ORs), *p*‐values, and 95% CIs to demonstrate the degree of association.

Univariate logistic regression was applied to explore associations between potential risk factors and LSD status at both farm and individual animal levels. Crude ORs with 95% CIs and *p*‐values were estimated. Variables with a *p*‐value < 0.05 in the univariate analysis were considered for multivariate modeling.

Multivariate logistic regression was conducted using a backward stepwise selection approach to identify independent risk factors associated with LSD occurrence. Variables with *p*‐values > 0.05 were sequentially removed to derive the most accurate model. Because multiple animals were sampled from the same farm, cluster‐robust standard errors were applied at the farm level to account for intracluster correlation. The final multivariate models for both farm and animal levels included only variables that remained statistically significant at *p* < 0.05. Adjusted odds ratios (aORs) with 95% CIs were calculated to assess the independent effects of each predictor while controlling for potential confounders. The goodness‐of‐fit of the final models was evaluated using the Hosmer–Lemeshow test and area under the curve (AUC). STATA 13 (StataCorp LLC, College Station, TX, USA) was used for all statistical analyses.

### 2.6. Spatial Analysis

Spatial analysis was conducted to evaluate the clustering of LSD‐affected farms across the study area. The geographical distribution of case and control farms was mapped using ArcGIS 10.8 (Esri, Redlands, CA, USA). GPS coordinates were recorded for 85 unions, the lowest administrative units within the 9 subdistricts (upazilas) of the three study districts from which farms were sampled, and spatial autocorrelation analysis was performed to assess clustering patterns at the union level. Spatial autocorrelation was assessed using Moran’s I statistic, computed in ArcGIS 10.8 using the Spatial Statistics Tools with an inverse‐distance spatial weights matrix to define neighborhood relationships between union centroids. This analysis provided insights into the spatial epidemiology of LSD and potential hotspot regions for targeted interventions.

### 2.7. Ethical Consideration

The study was conducted under the routine responsibilities of the Government Veterinary Authority, Barishal, Bangladesh, with approval from the District Livestock Office, Department of Livestock Services, Bangladesh (Memo no: 33.01.0000.16.004.22−458; date: 10 August 2023). Written informed consent was obtained from all farmers prior to data collection, ensuring voluntary participation. The confidentiality of all collected data was strictly maintained, and participants were informed of their right to withdraw from the study at any time without consequence. Formal Institutional Review Board (IRB) approval was not required.

## 3. Results

### 3.1. Overall Prevalence

The overall farm‐ and animal‐level prevalence of LSD in cattle was 15.4% (194/1260) and 13.1% (867/6624), respectively. Significant risk differences were found between young and adult cattle, local and crossbred breeds, and female and male cattle. Specifically, young cattle showed a higher prevalence (8.7%; 95% CI: 7.9–9.5) compared to adults (4.4%), local breeds had higher prevalence (7.7%; 95% CI: 1.4–3.0) compared to crossbreeds (5.5%), and females had higher prevalence (7.3%; 95% CI: 0.7–2.3) compared to males (5.8%) (Table 1).

**Table 1:** Prevalence difference of lumpy skin disease (LSD) by age, breed, and sex in cattle (*N* = 6624).

Comparison	1st category (%), 95% CI	2nd category (%), 95% CI	Risk difference (%), 95% CI	Relative risk (RR) %	*p* value
Young vs. adult	8.7 (315/3622), 7.9–9.5	4.4 (512/3002), 3.9–4.9	4.3, 3.5–5.1	2.0	<0.01
Local vs. cross	7.7 (510/6624), 7.0–8.4	5.5 (357/6624), 4.9–6.1	2.2, 1.4–3.0	1.4	<0.01
Female vs. male	7.3 (428/5841), 6.5–8.1	5.8 (439/5841), 5.1–6.5	1.5, 0.7–2.3	1.3	0.01

### 3.2. Spatial Variation in LSD Outbreak Farms

**Figure 3:**
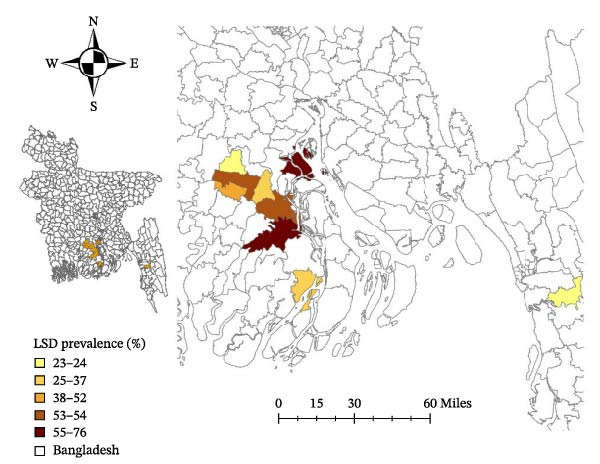
Subdistrict–level prevalence of lumpy skin disease (LSD) in cattle in Southern Bangladesh (2023). LSD prevalence (%) by subdistrict is displayed using a choropleth map classified by natural breaks (Jenks) into five categories: 23%–24%, 25%–37%, 38%–52%, 53%–54%, and 55%–76%. Darker shading represents higher prevalence. Administrative boundaries for Bangladesh are shown for spatial reference. A scale bar and north arrow are included for orientation.

Farm‐level LSD prevalence varied markedly between subdistricts (Table 2, Figure 3). The prevalence of LSD demonstrated significant spatial variation across subdistricts. The highest prevalence was observed in Barishal (73.4%), followed by Patuakhali (17.5%) and Chattogram (9.1%). Among the subdistricts, Bakerganj in Barishal recorded the highest odds of LSD outbreaks (OR = 10.6, 95% CI: 4.9–23.1, *p* < 0.001), followed by Hijla (OR = 7.6, 95% CI: 3.6–16.0, *p* < 0.001) and Dashmina in Patuakhali (OR = 1.9, 95% CI: 1.1–3.6, *p* = 0.04), relative to Chandanaish in Chattogram, which had the lowest prevalence (23.5%). Chandanaish was chosen as the reference subdistrict due to its lower LSD prevalence. To account for multiple comparisons, the Bonferroni correction was applied, reducing the risk of Type I errors.

**Table 2:** Spatial variation of lumpy skin disease (LSD) outbreaks among cattle in Southern Bangladesh, 2022–2023 (*N* = 1260).

District	Prevalence (%)	Subdistrict	LSD (+) (%)	OR	95% CI	*p* value (*χ*‐square)
Chattogram	9.1	Chandanaish	23.5	Ref	—	—
Patuakhali	17.5	Dashmina	37.2	1.9	1.1–3.6	0.04
Barishal	73.4	Agailjhara	22.6	0.9	0.5–1.8	0.88
		Babuganj	37.2	1.9	1.1–3.6	0.04
		Bakerganj	76.5	10.6	4.9–23.1	<0.001
		Banaripara	52.0	3.5	2.1–6.0	<0.001
		Barishal Sadar	53.2	3.7	2.3–5.9	<0.001
		Hijla	70.0	7.6	3.6–16.0	<0.001
		Wazirpur	54.0	3.8	1.9–7.7	<0.001

### 3.3. Clinical Outcomes of Cattle Affected by LSD

Among the 315 clinically affected cattle from farms experiencing active LSD outbreaks at the time of data collection, 75.9% recovered, 12.1% were sold, 11.1% remained ill, and 0.9% died (case fatality rate [CFR]), with recovery rates higher in adults and females than in young and male cattle, respectively (Table 3). The “sold” category refers to distress sales due to illness, where cattle were sold in poor health. This may introduce bias in the CFR, potentially overestimating mortality, as animals in poorer health are more likely to be sold.

**Table 3:** The clinical outcome of cattle infected with LSD by age, breed, sex and BCS (*N* = 315).

Variables	Categories	Outcome of the affected animal, % (*n*)			
		Death	Recovered	Sold	Still ill
Age	Young (1–24 m)	1.4 (3)	72.4 (155)	13.6 (29)	12.6 (27)
	Adult (>24 m)	0.0 (0)	83.2 (84)	8.9 (9)	7.9 (8)
Breed	Local	3.0 (3)	78.2 (79)	3.0 (3)	15.8 (16)
	Cross	0.0 (0)	74.8 (160)	16.4 (35)	8.9 (19)
Sex	Female	0.6 (1)	83.4 (151)	6.6 (12)	9.4 (17)
	Male	1.5 (2)	65.7 (88)	19.4 (26)	13.4 (18)
BCS	BCS 1 & 2	0.0 (0)	75.0 (33)	11.4 (5)	13.6 (6)
	BCS 3	1.7 (3)	76.1 (137)	12.8 (23)	9.4 (17)
	BCS 4 & 5	0.0 (0)	75.8 (69)	11.0 (10)	13.2 (12)
	Total	0.9 (3)	75.9 (239)	12.1 (38)	11.1 (35)

### 3.4. Risk Factors for LSD at the Farm Level

In the univariate analysis, the presence of stagnant water bodies near farms was significantly associated with an increased odds of LSD (OR = 15.8, 95% CI: 2.2–115.3). However, the very large OR and wide confidence interval suggest substantial uncertainty around this estimate, and the true effect may differ from the observed value. This could be due to confounding factors or measurement issues, such as inaccurate reporting of water sources or unmeasured environmental variables. Similarly, farms with mud flooring had substantially higher odds of LSD (OR = 9.1, 95% CI: 3.4–24.6) compared to those with concrete flooring. The disposal of manure in ponds was also associated with a greater risk of LSD (OR = 1.7, 95% CI: 1.1–2.6). The use of mosquito coils and chemical sprays for insect control was significantly associated with LSD occurrence (OR = 1.8, 95% CI: 1.3–2.7, and OR = 2.2, 95% CI: 0.9–5.6). However, reverse causation must be considered, as farms with LSD outbreaks may be more likely to apply insecticides. Future analyses should address lagging exposure or clearly note the limits of causality in these associations. Additionally, in farms with extensive management practices, perfect separation occurred (20/20 cases), which may lead to instability in regression models. This issue will be addressed using Firth logistic regression, exact methods, or by collapsing categories (Table 4).

**Table 4:** Risk factors for suspected LSD at farm level in Southern Bangladesh, 2023.

Variable	Case *n* (%)	Control *n* (%)	Univariate OR (95% CI, *p*)	Multivariate aOR (95% CI, *p*)
Nearest cattle farm				
Within 50 m	57 (28.8)	146 (71.9)	Ref.	—
>50 m	137 (22.4)	475 (77.6)	1.4 (0.9–1.9, 0.10)	—
Stagnant water bodies near farm				
No	1 (2.1)	47 (97.9)	Ref.	—
Yes	193 (25.2)	574 (74.8)	15.8 (2.2–115.3, 0.01)	—
Distance of water bodies from farm				
Within 50 m	69 (24.0)	219 (76.0)	Ref.	—
>50 m	124 (25.9)	355 (74.1)	1.1 (0.8–1.7, 0.55)	—
Bush/jungle within 500 m				
No	138 (29.1)	336 (70.9)	Ref.	—
Yes	56 (16.4)	285 (83.6)	0.5 (0.3–0.7, <0.01)	—
Floor type of animal house				
Concrete	5 (8.5)	54 (91.5)	Ref.	—
Brick with straw	86 (23.8)	275 (76.2)	3.4 (1.3–8.7, 0.010)	—
Cement	49 (17.9)	225 (82.1)	2.4 (0.9–6.2, 0.080)	—
Mud	48 (45.7)	57 (54.3)	9.1 (3.4–24.6, <0.01)	—
Manure disposal practice				
Disposal in pits	117 (20.3)	459 (79.7)	Ref.	—
Scattered disposal	15 (22.1)	53 (77.9)	1.1 (0.6–2.0, 0.73)	1.1 (0.6–2.2, 0.77)
Pond disposal	42 (30.7)	95 (69.3)	1.7 (1.1–2.6, 0.01)	**2.1 (1.3–3.3, 0.01)**
Cleaning strategy				
Using hose pipe	31 (15.4)	170 (84.6)	Ref.	—
Swiping with water	85 (21.9)	304 (78.2)	1.5 (0.9–2.4, 0.06)	—
Removing manure only	68 (36.7)	118 (63.4)	3.1 (1.9–5.1, <0.01)	—
Frequency of cleaning cattle house				
Once daily	35 (20.1)	139 (79.9)	Ref.	—
Twice daily	95 (23.8)	304 (76.2)	1.2 (0.8–1.9, 0.33)	—
Thrice daily	63 (27.6)	165 (72.4)	1.5 (0.9–2.4, 0.08)	—
After one or more day	0 (0)	13 (100)	—	—
Vector control measures				
Mosquito net	58 (15.5)	316 (84.5)	Ref.	—
Mosquito Coil	93 (25.4)	273 (74.6)	1.8 (1.3–2.7, 0.01)	2.0 (0.8–5.3,0.14)
Chemical spray	7 (29.2)	17 (70.8)	2.2 (0.9–5.6, 0.08)	**1.9 (1.3–2.7, 0.01)**
Communal grazing				
No	68 (17.9)	313 (82.2)	Ref.	—
Yes	126 (29.0)	308 (71.0)	1.88 (1.35−2.63, <0.01)	**1.7 (1.2–2.5, 0.01)**
Grazing land type				
Marshy low land	29.8 (25)	59 (70.2)	Ref.	—
In paddy field	14 (56)	11 (44)	3.0 (1.2–7.5, 0.02)	—
In dry low land	81 (25.2)	241 (74.8)	0.7 (0.5–1.3,0.39)	—

*Note:* Percentages are presented by row. “Not estimable” indicates zero observations in comparator group. Multivariable model included variables with *p* < 0.05 in univariate analysis. Adjusted ORs are reported only for variables retained in the final multivariable model after stepwise backward elimination. Bold values indicate statistically significant results at *p* < 0.05 level.

Abbreviations: CI, confidence interval; OR, odds ratio.

Multivariate analysis confirmed several risk factors for LSD. Compared with farms disposing of manure away from water bodies, farms that disposed of manure in ponds had 2.1 times higher odds of LSD (aOR = 2.1, 95% CI: 1.3–3.3). Compared with the use of mosquito nets, the use of chemical sprays for insect control remained a significant risk factor (aOR = 1.9, 95% CI: 1.3–2.7). Communal grazing was also associated with increased odds of LSD (aOR = 1.7, 95% CI: 1.2–2.5), suggesting a heightened risk due to shared exposure. Goodness‐of‐fit testing revealed that the model was appropriate for the data (Pearson chi2(10) = 24.34). The AUC for the logistic regression model was 0.6364, indicating moderate predictive ability of the model in differentiating between LSD cases and noncases (Table 4).

### 3.5. Animal‐Level Risk Factors for LSD

In the univariate analysis, young cattle (7–24 months) (OR = 1.4, 95% CI: 1.1–1.8) and calves (0–6 months) (OR = 1.5, 95% CI: 1.0–2.2) exhibited significantly increased odds of contracting LSD compared to mature cattle (>24 months). Local‐breed cattle had a significantly higher risk of LSD, with 5.7 times greater odds (OR = 5.7, 95% CI: 4.3–7.5) compared to crossbreeds. Among female cattle, milking cows exhibited a notably lower risk of infection (OR = 0.4, 95% CI: 0.2–0.8) when compared to dry cows. Cattle with poor body condition scores (BCS 1 & 2) were 4.2 times more likely to develop LSD (OR = 4.2, 95% CI: 2.5–7.0) than those with good body condition (BCS 4 & 5). Additionally, cattle that participated in communal grazing before the outbreak occurred in their herd had 14.3 times higher odds of contracting LSD (OR = 14.3, 95% CI: 10.5–19.6).

Multivariate analysis confirmed that local‐breed cattle remained at significantly higher risk (aOR = 2.5, 95% CI: 1.4–4.5). Cattle with poor body condition (BCS 1 & 2) had twice the odds of developing LSD (aOR = 2.2, 95% CI: 0.9–5.9). The strongest independent risk factor identified was communal grazing prior to the onset of the disease, with cattle having 9.9 times higher odds of infection (aOR = 9.5, 95% CI: 5.2–17.3) (Table 5). Goodness‐of‐fit testing indicated that the model was appropriate for the data (Pearson chi2(6) = 19.97). The Area Under the Curve (AUC) for the model was 0.8109, reflecting good predictive ability in differentiating LSD cases.

**Table 5:** Determination of risk factors for LSD at animal level in Southern Bangladesh, 2023.

Variable	Case *n* (%)	Control *n* (%)	Univariate OR (95% CI, *p*)	Multivariate aOR (95% CI, *p*)
Age				
Mature (<24 m)	101 (21.7)	365 (78.3)	Ref.	—
Young (7–24 m)	162 (28.3)	411 (71.7)	1.4 (1.1–1.8,0.02)	—
Calf (0–6 m)	52 (29.3)	125 (70.6)	1.5 (1.0–2.2,0.04)	—
Breed				
Cross	101 (13.3)	656 (86.7)	Ref.	—
Local	214 (46.6)	245 (53.4)	5.7 (4.3–7.5), < 0.01	**2.5 (1.4–4.5, <0.01)**
Sex				
Male	134 (24.5)	412 (75.5)	Ref.	—
Female	181 (27.0)	489 (73.0)	1.1 (0.9–1.5, 0.32)	—
Production stage of female cattle				
Dry cow	18 (34.0)	35 (66.0)	Ref.	—
Heifer	93 (36.5)	162 (63.5)	1.1 (0.6–2.1, 0.72)	—
Milking cow	35 (17.3)	167 (82.7)	0.4 (0.2–0.8, 0.02)	—
Pregnant cow	35 (21.9)	125 (78.1)	0.5 (0.3–1.1, 0.08)	—
BCS				
BCS 4 & 5	91 (23.4)	298 (76.6)	Ref.	—
BCS 3	180 (24.0)	569 (76.0)	1.0 (0.8–1.4, 0.81)	1.2 (0.7–2.1, 0.51)
BCS 1 & 2	44 (56.4)	34 (43.6)	4.2 (2.5–7.0, <0.02)	**2.2 (1.2–4.2, 0.02)**
Communal grazing				
No	197 (67.7)	94 (32.3)	Ref.	—
Yes	118 (12.8)	807 (87.2)	14.3 (10.5–19.6, < 0.02)	**9.4 (5.2** **–17.3, <0.01)**

*Note:* Percentages are presented by row. “Not estimable” indicates zero observations in comparator group. Multivariable model included variables with *p* < 0.05 in univariate analysis. Adjusted ORs are reported only for variables retained in the final multivariable model after stepwise backward elimination. Bold values indicate statistically significant results at *p* < 0.05 level.

Abbreviations: CI, confidence interval; OR, odds ratio.

In our study, communal grazing showed significant association with LSD status at both the farm and individual levels, although the magnitude of association differed between the two levels. This indicates that the practice influences disease occurrence not only through herd management but also through individual animal exposure.

### 3.6. Spatial Autocorrelation

The spatial autocorrelation analysis showed a clustering pattern in the prevalence data, as evidenced by a Moran’s index value of 0.25 (where 0 indicates randomness). However, this clustering was not statistically significant, with a *p*‐value of 0.4095.

## 4. Discussion

LSD has emerged as a significant threat to cattle health and livestock production in Bangladesh, posing substantial economic and epidemiological challenges. In our study, we identified three principal findings: At the farm level, LSD prevalence was significantly associated with communal grazing, where cattle from different herds shared grazing areas, facilitating disease transmission. At the animal level, cattle in poor body condition had a higher likelihood of contracting LSD compared to those in better condition, and local‐breed cattle exhibited a higher prevalence of LSD compared to crossbred cattle. These findings highlight the critical role of both farm management practices and animal characteristics in the transmission of LSD.

A study of Barishal District, Bangladesh, reported in 2023 that calves aged 0–6 months had a maximum prevalence of 33.84%, while older age groups showed lower infection rates [24], which supports our current finding. This increased susceptibility in younger cattle is likely due to their immature immune systems, which are less effective at combating infections [25]. Additionally, management practices that facilitate close contact among animals may contribute to higher exposure rates to infected animals or vectors, further increasing disease prevalence in this age group [26]. Our study also demonstrated that younger cattle had significantly higher odds of LSD compared to mature cattle. These findings reinforce the notion that age‐related immune responses and management factors play critical roles in LSD susceptibility. Conversely, some studies have reported no significant difference in LSD prevalence between age groups. For instance, research in Ethiopia found that LSD affected all cattle, regardless of age, with both local and crossbreeds being susceptible [27]. This suggests that factors other than age, such as breed susceptibility, environmental conditions, and management practices, may play a more critical role in disease transmission.

**Figure 4:**
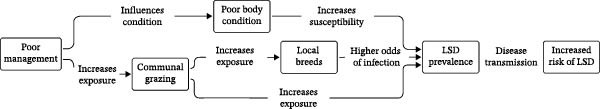
Directed acyclic graph (DAG) illustrating the relationships between farm management practices (poor management, communal grazing), animal body condition, and breed‐specific factors in the transmission of lumpy skin disease (LSD). The graph shows how these factors might influence exposure and susceptibility, potentially confounding the relationship between breed and disease prevalence, leading to increased risk of LSD in cattle.

We observed that local cattle breeds had a higher prevalence of LSD compared to crossbreeds, which contrasts with some literature suggesting that local breeds possess greater resistance to endemic diseases due to adaptive immunity. A study in Chattogram, Bangladesh, reported a lower infection rate of LSD in local cattle compared to crossbreeds [13]. However, our findings showed that local breeds had 2.5 times higher odds of LSD compared to crossbreeds, with this result remaining significant in the multivariate analysis. Similar findings have been reported in India, where local breeds appeared more frequently affected by LSD; however, this increased susceptibility has been attributed largely to the conditions under which these animals are kept, including malnutrition with impaired cellular immunity, poorer management, and more limited access to veterinary care in rural areas [28]. Conversely, studies from Bangladesh found that local breeds were more resilient to LSD, likely due to better adaptation to local environmental conditions [13]. The higher infection risk in local cattle in Bangladesh is likely linked to contextual factors such as communal grazing practices, which increase exposure to infected animals and vectors, and inadequate veterinary care [13]. While breed‐specific immune differences may play a role, these findings emphasize the importance of management practices and grazing exposure that influence disease risk rather than implying innate breed susceptibility [3] (Figure 4).

In this study, vector control measures—such as mosquito nets, mosquito coils, and chemical sprays—were evaluated for their effectiveness in controlling LSD. LSDV is transmitted primarily through the mechanical action of biting arthropods, notably *Stomoxys calcitrans* and *Aedes* mosquitoes, which are prevalent in the humid climate of Southern Bangladesh [29]. Although mosquito nets were associated with reduced LSD risk, conventional nets provided limited protection against stable flies, which are among the main vectors [29]. Insecticide‐treated or fine‐mesh nets may therefore offer improved protection by targeting a broader range of vectors [30]. In contrast, short‐acting measures such as mosquito coils and chemical sprays appear less effective [31], emphasizing the need for integrated vector management approaches, including housing improvement, manure management, and environmental modification, to reduce breeding sites. This association should be interpreted cautiously as increased insecticide use may reflect reactive application following outbreak detection rather than a true causal effect. Despite their lower effectiveness, mosquito coils and chemical sprays remain widely used due to affordability and availability [32]. However, their repeated application raises environmental and health concerns and underscores the need for more sustainable and evidence‐based control measures. Notably, a “no‐control” group could not be included as all farms had adopted at least one protective measure in response to the high burden of LSD in the region. Communal grazing and watering points have been identified as significant risk factors for the transmission of LSDV in cattle. Through communal grazing, infected animals shed the virus in saliva, nasal secretions, and lesions, contaminating the shared pastures [33]. Therefore, the virus infects healthy animals when they come in contact with the pastures during grazing. In rural Bangladesh, communal grazing remains prevalent due to land constraints and traditional livestock management practices [34]. Smallholder farmers often lack access to private grazing lands, compelling them to rely on shared pastures where cattle from multiple farms intermingle, increasing the risk of LSDV transmission [34]. Additionally, studies have reported that local breeds in rural areas frequently face constraints such as poorer management practices and reduced access to veterinary services, which may further increase their vulnerability to infection [35]. In our study, the higher prevalence of LSD in local breeds might therefore reflect greater exposure to communal grazing and environmental risk factors, in combination with these management‐ and service‐related constraints, rather than a lack of inherent resistance.

A study conducted in Nakuru County, Kenya, demonstrated that farms practicing communal grazing had higher odds of LSD outbreaks, suggesting that the shared‐pasturing of cattle from different herds facilitates the spread of the virus [20]. Similarly, research in Northern Egypt found that communal grazing areas contributed to increased LSD seroprevalence, highlighting the role of shared pastures in disease dissemination [36]. The same study in Kenya highlighted that farms with limited veterinary support experienced higher rates of LSD [20]. Notably, this association is not exclusive to LSD but extends to various infectious diseases as insufficient veterinary intervention compromises disease prevention, timely diagnosis, and effective management. The presence of vector populations, such as biting flies, is influenced by environmental conditions, and local breeds in rural settings might have greater exposure to these vectors [37]. Research has shown that vector control is essential in managing LSD outbreaks, particularly in areas with high vector densities [26].

We found a slightly higher prevalence of LSD in female cattle compared to males, which is consistent with findings from Chattogram, Bangladesh, where females were at greater risk of infection [13]. This increased susceptibility may be attributed to physiological factors as pregnancy and lactation can induce immunosuppression, making female cattle more vulnerable to infections. Studies indicate that immune modulation during these stages can weaken the body’s defense against pathogens [38]. Furthermore, female cattle are typically retained longer for breeding and milk production, increasing their cumulative exposure to infection sources. Traditional management practices, where female cattle are housed in closer proximity for milking and reproduction, might further facilitate disease transmission. Given that LSD is primarily spread through biting insects, prolonged exposure to vectors in confined spaces can elevate infection risks [39]. However, our study found that milking cows had a significantly lower risk of LSD compared to dry cows. As milking cows may receive better nutrition and veterinary care, this could enhance immune function and lower disease susceptibility. Stratified analyses by farm management intensity, such as comparing intensive versus extensive systems, could help to better understand the influence of farm practices on this outcome. Some studies have reported no significant sex‐based differences in LSD prevalence, suggesting that other factors—such as breed, environmental conditions, and farm management practices—might also influence disease occurrence [40]. Additionally, reverse causation should be considered as poor body condition (BCS) in dry cows could be a consequence of illness rather than a predisposing factor, and prospective study designs could clarify the causal relationships between body condition and disease risk [41].

Body condition is another animal risk factor that plays a significant role in susceptibility to LSD. Cattle with poor BCSs were 2.2 times more likely to develop LSD compared to those with good body condition, and this association was found significant in multivariate analysis. Poor body condition is often associated with malnutrition, chronic illness, or high parasite loads, all of which can weaken immune responses and increase disease susceptibility [39]. Studies have shown that cattle with compromised health status are more prone to vector‐borne diseases, including LSD, due to their reduced ability to mount effective immune responses [42]. However, reverse causation should be considered as poor body condition could also result from illness rather than being a predisposing factor for LSD. Prospective study designs would help clarify the temporal relationship between the body condition and the development of LSD, providing more insights into the direction of causality.

**Figure 5:**
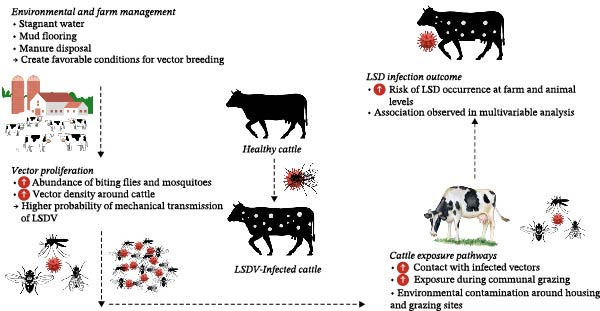
Conceptual pathway linking environmental conditions, vector activity and cattle exposure leading to LSD infection. This diagram summarizes how key environmental and management conditions identified in the study, including stagnant water near farms, mud flooring and manure disposal in ponds, promote vector breeding. Increased populations of biting flies and mosquitoes heighten the likelihood of mechanical transmission of LSDV. Cattle become exposed through contact with infected vectors and through communal grazing, resulting in a higher risk of LSD infection as demonstrated by the multivariable analysis.

In our study, several farm‐level management factors were found significantly associated with the occurrence of LSD in cattle (Figure 5). Notably, the presence of stagnant water bodies near farms was identified as a significant risk factor, with an odds ratio of 15.8. Stagnant water serves as an ideal breeding ground for insect vectors, particularly mosquitoes and biting flies, which are implicated in the transmission of LSDV. This finding aligns with previous research highlighting the role of arthropod vectors in the epidemiology of LSD [43].

Flooring type in cattle housing was another significant factor influencing the risk of LSD. Specifically, mud flooring was associated with an increased risk compared to that of concrete flooring. This association is likely due to the fact that mud floors can harbor moisture and organic matter, creating an environment conducive to the breeding of insect vectors such as mosquitoes and flies, which are known to transmit LSDV (Figure 5). Additionally, mud flooring can lead to poor hygiene conditions, facilitating the persistence and transmission of pathogens. Improved housing conditions, including the use of concrete or other easily cleanable surfaces, can reduce the risk of LSD by minimizing contact with contaminated surfaces and reducing vector breeding sites. A study assessing the impact of housing systems and environmental features on beef cattle welfare supports this, indicating that flooring type can influence the microclimate and overall health of cattle [44].

We found that the farms that disposed manure in ponds had higher odds of LSD, likely due to the role of organic waste in attracting insect vectors. A study on the clinical management of LSD emphasizes the importance of proper manure disposal to prevent vector attraction and subsequent disease spread [45].

Beyond risk factor assessment, this study identified significant spatial heterogeneity in LSD prevalence, with Barishal exhibiting the highest burden, particularly in Bakerganj and Hijla, suggesting potential epidemiological hotspots. The high LSD prevalence in certain subdistricts may be attributed to regional differences in housing system, vector populations, and cattle movement patterns [12]. High farm density can facilitate rapid disease spread, while abundant vector populations, such as biting flies, enhance transmission efficiency. Frequent cattle movements, especially in trading hubs, further contribute to the dissemination of the virus. These findings highlight the need for geographically targeted interventions, such as intensified surveillance and localized vector control efforts, in high‐risk areas. Implementing biosecurity measures tailored to specific regional risks can effectively mitigate LSD outbreaks.

## 5. Limitations

Despite several strengths of this study, it has several limitations as well. The convenience sampling method limits the generalizability of the study as only farms in accessible subdistricts were included. This nonrandom sampling approach can introduce selection bias and might not represent the full diversity of outbreak areas across Southern Bangladesh. The unequal number of case and control farms and animals may have reduced statistical power and increased uncertainty in some estimates, particularly in multivariable analyses. These limitations should be considered when interpreting the study findings. A major limitation is the reliance on clinical diagnosis without laboratory confirmation of LSD cases, which increases the risk of misclassification, particularly in mild or atypical cases. This could lead to under‐ or overestimation of disease prevalence. Underreporting of LSD, especially in rural and small‐scale farms with limited documentation, hindered data collection and reduced the availability of confirmed cases. The lack of accurate data on outbreak onset and duration due to the absence of a proper data recording system also limited our ability to track disease spread. Additionally, the ongoing nature of the outbreak beyond the study period prevented a full assessment of its temporal dynamics.

Additionally, farm‐level biosecurity measures were highly variable, making it challenging to control for confounding factors in disease transmission analysis.

The clinical variability of LSD, which includes both symptomatic and asymptomatic infections, further complicates case identification. High morbidity and mortality in clinically affected cattle also limited access to comprehensive data from affected farms. Moreover, the cross‐sectional design of the study only allows for the identification of associations rather than causation. Recall bias is another concern as structured interviews relied on farmers’ recollection of past outbreaks and management practices. Environmental and climatic factors, such as vector density, temperature, humidity, and rainfall, were not incorporated, even though they are critical determinants of LSD transmission. Furthermore, although a few animals were vaccinated, the vaccination status was not fully addressed, leaving the effect of immunization on disease susceptibility uncertain.

To address these limitations, future studies should incorporate laboratory‐confirmed diagnostics, such as PCR or serological testing, to improve case‐identification accuracy. Longitudinal study designs are recommended to establish causal relationships between risk factors and LSD occurrence. Entomological surveillance should measure vector density and species composition, while meteorological data could improve understanding of seasonal disease dynamics.

From an intervention perspective, vaccination campaigns should target high‐risk groups, particularly young and local‐breed cattle. Farm management practices, including proper manure disposal and improved housing, should be emphasized. Vector control strategies should move beyond mosquito coils and chemical sprays toward more effective measures, such as insecticide‐treated nets and environmental modifications to reduce breeding sites. Biosecurity measures, including controlled or rotational grazing, should be implemented to minimize contact between infected and healthy animals. Strengthening veterinary outreach programs and establishing regular surveillance for LSD at farm level should improve early detection, facilitate rapid response, and support the implementation of risk‐based vaccination and vector‐control strategies. At the policy level, zoning strategies and subsidies for vaccination, housing improvements, and vector control can support smallholder farmers in implementing effective LSD prevention measures.

## 6. Future Directions

Future research on lumpy skin Disease (LSD) should focus on longitudinal studies to establish causal relationships between risk factors and disease progression, alongside laboratory‐confirmed diagnostics (e.g., PCR or serological testing) to improve case accuracy. Incorporating environmental factors such as vector density, temperature, and rainfall through entomological and meteorological surveillance will enhance understanding of seasonal dynamics. More effective vector control strategies, such as insecticide‐treated nets and environmental modifications, should be explored. Spatial autocorrelation and hotspot mapping can identify high‐risk areas for targeted interventions, while research on vaccine efficacy and cattle immunity will help develop more effective vaccination programs. Improving data recording systems for real‐time surveillance, strengthening veterinary outreach, and exploring policy interventions such as vaccination subsidies and vector control can significantly mitigate LSD’s impact in affected regions.

## 7. Conclusion

This study provides critical insights into the epidemiology of LSD in Southern Bangladesh. The findings identify several key risk factors, including manure disposal in ponds, communal grazing, and the vulnerability of local cattle breeds and poorly conditioned animals. These factors underscore the need for targeted vaccination, improved manure management, and community‐level biosecurity interventions to significantly reduce LSD transmission in endemic zones. Effective control measures should focus on high‐risk farms, with emphasis on risk‐based vaccination, enhanced biosecurity, and more robust vector control methods. The study’s results also point to the importance of integrating farm‐level management and environmental conditions into LSD surveillance systems to better predict outbreaks. Moving forward, longitudinal studies and environmental surveillance are essential for refining control strategies and informing policy development aimed at mitigating the impact of LSD on livestock health and productivity in the region.

## Funding

This study was funded by the Australian Research Council (Grant FL240100037).

## Conflicts of Interest

The authors declare no conflicts of interest.

## Data Availability

The data that support the findings of this study are available upon request from the corresponding author. The data are not publicly available due to privacy restrictions.
